# Structural, optical and microwave dielectric properties of Ba(Ti_1−x_Sn_x_)_4_O_9_, 0 ≤ x ≤ 0.7 ceramics

**DOI:** 10.1038/s41598-021-97584-x

**Published:** 2021-09-09

**Authors:** Asad Ali, Sarir Uddin, Madan Lal, Abid Zaman, Zafar Iqbal, Khaled Althubeiti

**Affiliations:** 1 Department of Physics, Riphah International University, Islamabad, 44000 Pakistan; 2 Department of Physics, Government Post Graduate College, Nowshera, KP Pakistan; 3 Department of Physics, Government Degree College Hayatabad, Peshawar, 25000 Pakistan; 4 Department of Physics, Akal College of Basic Sciences, Eternal University, Sirmour, H.P 173101 India; 5 Department of Chemistry, College of Science, Taif University, Taif, 21944 Saudi Arabia

**Keywords:** Materials science, Nanoscience and technology, Physics

## Abstract

Sn-doped BaTi_4_O_9_ (BT4) dielectric ceramics were prepared by a mixed oxide route. Preliminary X-ray diffraction (XRD) structural study shows that the ceramic samples have orthorhombic symmetry with space group (Pnmm). Scanning electron microscopy (SEM) shows that the grain size of the samples decreases with an increase in Sn^4+^ content. The presence of the metal oxide efficient group was revealed by Fourier transform infrared (FTIR) spectroscopy. The photoluminescence spectra of the ceramic samples reported red color ~ 603, 604, 606.5 and 605 nm with excitation energy ~ 2.06, 2.05, 2.04 and 2.05 eV for Sn^4+^ content with x = 0.0, 0.3, 0.5, and 0.7, respectively. The microwave dielectric properties of these ceramic samples were investigated by an impedance analyzer. The excellent microwave dielectric properties i.e. high dielectric constant (ε_r_ = 57.29), high-quality factor (Q_*f*_ = 11,852), or low-dielectric loss (3.007) has been observed.

## Introduction

Dielectric ceramic materials are frequently used in modern telecommunication systems like satellite modules and cellular mobile phones. Layered-structured dielectric materials have been widely investigated for their potential use in ferroelectric random access memories (FeRAM) and piezoelectric devices. Ferroelectric and piezoelectric ceramic materials are broadly used in a range of applications such as sensors, actuators, transducers, pulse signal circuits, spacecraft, X-ray equipment, weapons, medical devices, and transportation. The dielectric component used in these devices is called dielectric resonators (DR). The optimum commercial dielectric properties of these ceramics included excellent dielectric constant (ε_r_), good quality factor (Q_*f*_) or low tangent loss (tan σ), and near to zero temperature coefficient at resonant frequency (τ_f_)^1–6^. For example, a dielectrically loaded antenna requires; ε_r_ from 20 to 85, (Q) ≥ 10,000, and τ_f_ close to zero^7,8^. Sometimes quality factor is expressed as the product of Q and *f*_o_ (resonant frequency) i.e. (Q × *f*_*o*_). The dielectric constant (ε_r_) and dielectric loss (tan σ) decrease with resonant frequency (*f*_o_), which suggests that electric dipoles and interfaces play an important role at the lower frequency range. The decrease in dielectric constant with resonant frequency can be explained based on Koops theory, reported by Praveena and Varma^9^. A relatively good dielectric constant is required for the miniaturization of devices and low tangent loss is required for noise reduction with zero ${\uptau }_{{\text{f }}}$, which is important for the temperature stability of the DR^2^. From the manufacturing point of view, it is very difficult to obtain compounds with all three optimum properties along with a low cost. Among oxides compounds, BaTi_4_O_9_ (BT4) is one of the dielectric materials that may be used in the microwave domain as first reported by Rase and Roy^10^. It has been recorded that BaTi_4_O_9_ ceramics have ε_r_ = 37.3, Q = 27,200 GHz and τ_f_ =  + 15 ppm/°C^11^. On the other side, wet chemical methods (i.e. sol–gel, co-precipitation, or hydrothermal) can be used for the preparation of titanates ceramics^12^. The optical, structural, microstructural, and microwave properties of BaTi_4_O_9_ ceramic samples with various dopant elements have been extensively studied at microwave frequency. Many researchers studied the effects of B site cation substitutions and dielectric properties of those samples affected by the substitution of larger ionic radius (Sn^4+^) for smaller ionic radius (Ti^4+^) ions^13–15^**.** In this way, Veenhuis et al. processed compounds with good dielectric properties which have been used in the field of advanced laser technology and optical storage devices^16^.

In the present time, BT4 has been investigated broadly because of its good Q_f_, high ε_r,_ and small τ_f_. Because used widely in microwave dielectric resonator applications, patch antenna, microwave telecommunication devices, etc. During the densification of these titanates at the very high sintering temperature, enhanced dielectric properties are observed due to the phase and compositional defects (fluctuations) (i.e. because of the partial reduction of Ti^4+^ to Ti^3+^ ion)^17^. The aim of the present work, to achieve a material with enhanced structural and dielectric properties for device application. In this report, we describe the synthesis (i.e.via the mixed oxide method) and the structural, optical, and microwave dielectric properties of Sn-doped BaTi_4_O_9_ ceramics. The calcined powders and sintered pellets obtained were characterized by XRD (X-ray diffraction), SEM (scanning electron microscopy), impedance analyzer, and FTIR (Fourier transform infra-red). The microwave dielectric properties of ceramic samples are discussed in terms of their physical and chemical characteristics.

## Materials and methods

The starting raw materials along with purity grades were: BaCO_3_ (Merck, Germany, 99.9%), TiO_2_ (Aldrich Chemical Company, Inc., U.S.A, 99.9%), and SnO_2_ (Strem, Chemicals, U.S.A, 99.9%) used to make the solid solutions of Ba(Ti_1−x_Sn_x_)_4_O_9_, 0 ≤ x ≤ 0.7 by using mixed oxides route. These raw materials were weighted according to stoichiometric ratio and mixed for 12 h in distilled water by using horizontal ball milling. Then the slurry was dried in a microwave oven at 100 °C for one day and calcined at 1100 °C for 3 h in a nickel crucible in the air atmosphere with a heating–cooling rate of 10 °C/min. The calcined powders were grinded for 60 min with a mortar and pistol manually to avoid agglomerations. Then pressed 0.6–0.8 gm of powder in cylindrical pellets of thickness 2 mm and diameter 10 mm by using a hydraulic press (CARVER, USA) with a pressure of 80 MPa. Thereafter, these green pellets were sintered at 1320 °C temperature in the open air for 4 h with a heating–cooling rate of 10 °C/min. The XRD patterns of the compounds were recorded at room temperature using an X-ray powder diffractometer (JDX-3532, JEOL, Japan) with Cu Ka radiation (k = 1.5405 Å) in a wide range of Bragg angles (20° ≤ 2θ ≤ 60°) at a scanning rate of 2 deg min^-1^. The experimental density of the samples was measured by Archimedes’ principle using a density meter (MD-3005, Germany). Scanning electron microscopy (SEM, JEOL 7600F) was used to study the microstructure of the dense pellets. The optical properties of these ceramics were done by using the Fourier transformation infrared spectroscopy (FTIR, Perkin-Elmer) and PL spectroscopy. Dielectric properties were measured at microwave frequencies by LCR meter (Agilent 4287 A).

## Results and discussions

### XRD analysis

**Figure 1:**
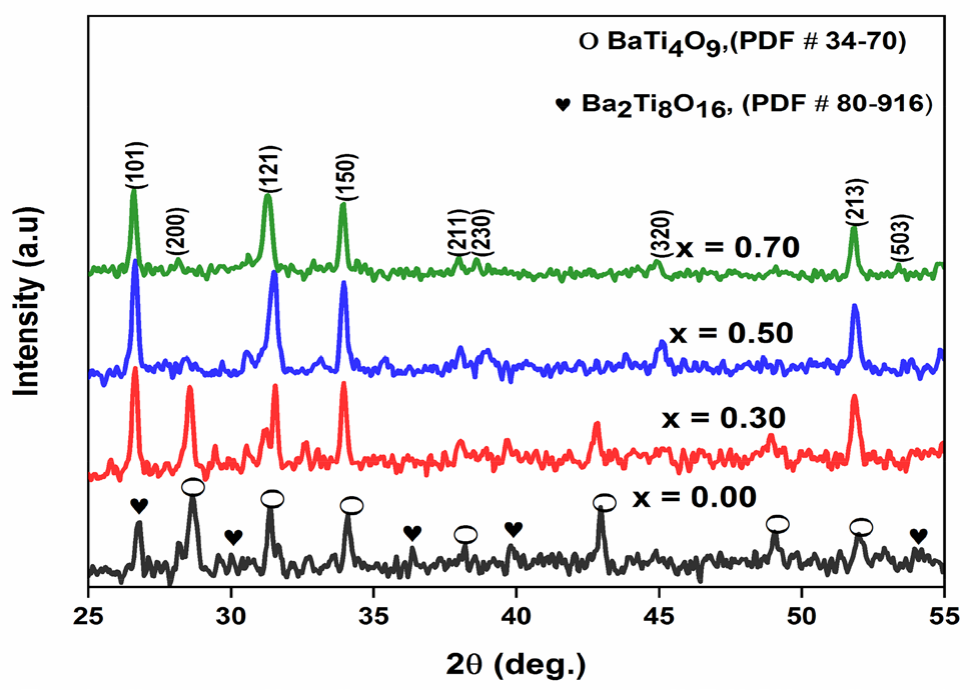
XRD pattern of Ba(Ti_1−x_Sn_x_)_4_O_9_, 0 ≤ x ≤ 0.7 ceramics.

Figure 1 represents the XRD patterns of Ba(Ti_1−x_Sn_x_)_4_O_9_, 0 ≤ x ≤ 0.7 ceramics at room temperature. The main peaks corresponding to (200), (121), (150), (211), (230), (320), (213), (503) planes are well-matched with PDF card number 34–70 of BaTi_4_O_9_ ceramics and have an orthorhombic structure with space group (Pnmm). Some of the secondary phases of Ba_2_Ti_8_O_18_ are detectable with PDF card number 0080–0916. It can be noted from Fig. 1 that the peaks shifted toward the lower 2θ values and representing the cell volume expansion with increasing the Sn^4^^+^ contents. This might be due to the inhomogeneity, micro-strain, or maybe due to the substitution of the relatively larger cation ions of Sn^4+^ (~ 0.69 Å) for the smaller cation of Ti^4+^ (~ 0.64 Å)^18^. The calculated lattice parameters (i.e. ‘a’, ‘b’, and ‘c’) increases with Sn^4+^ content. This increase in the lattice parameters may result in the phase transition from orthorhombic to a tetragonal structure. The average crystallite size of these samples was calculated by using Debye Scherer’s formula^19^. The observed average crystallite size and lattice parameters are given in Table 1.

1 $$  {\text{Crystallite}}\;{\text{size}},\;{\text{D}} = \frac{{0.9\lambda }}{{\beta {\text{Cos}}\theta }}  $$

where ‘θ’ is the brags angle, ‘λ’ is the wavelength of the incident radiation, and ‘β’ is the full-width of half-maximum (FWHM). The average crystallite size of these samples was lie in the range of 30–90 nm.

**Table 1:** Lattice parameters of Ba(Ti_1−x_Sn_x_)_4_O_9_, 0 ≤ x ≤ 0.7 ceramics obtained from XRD.

Composition (x)	Lattice parameters (Å)			Volume (Ǻ^3^)	Structure (space group)	Average crystallite size (D) nm
	a	B	c			
0.0	4.012	5.681	5.703	64.4	Orthorhombic (Pnmm)	90
0.3	4.024	4.024	4.045	65.1		50
0.5	4.036	4.036	4.057	65.7	Tetragonal (P4*mm*)	30
0.7	4.058	4.058	4.042	66.3		70

### Microstructure analysis

**Figure 2:**
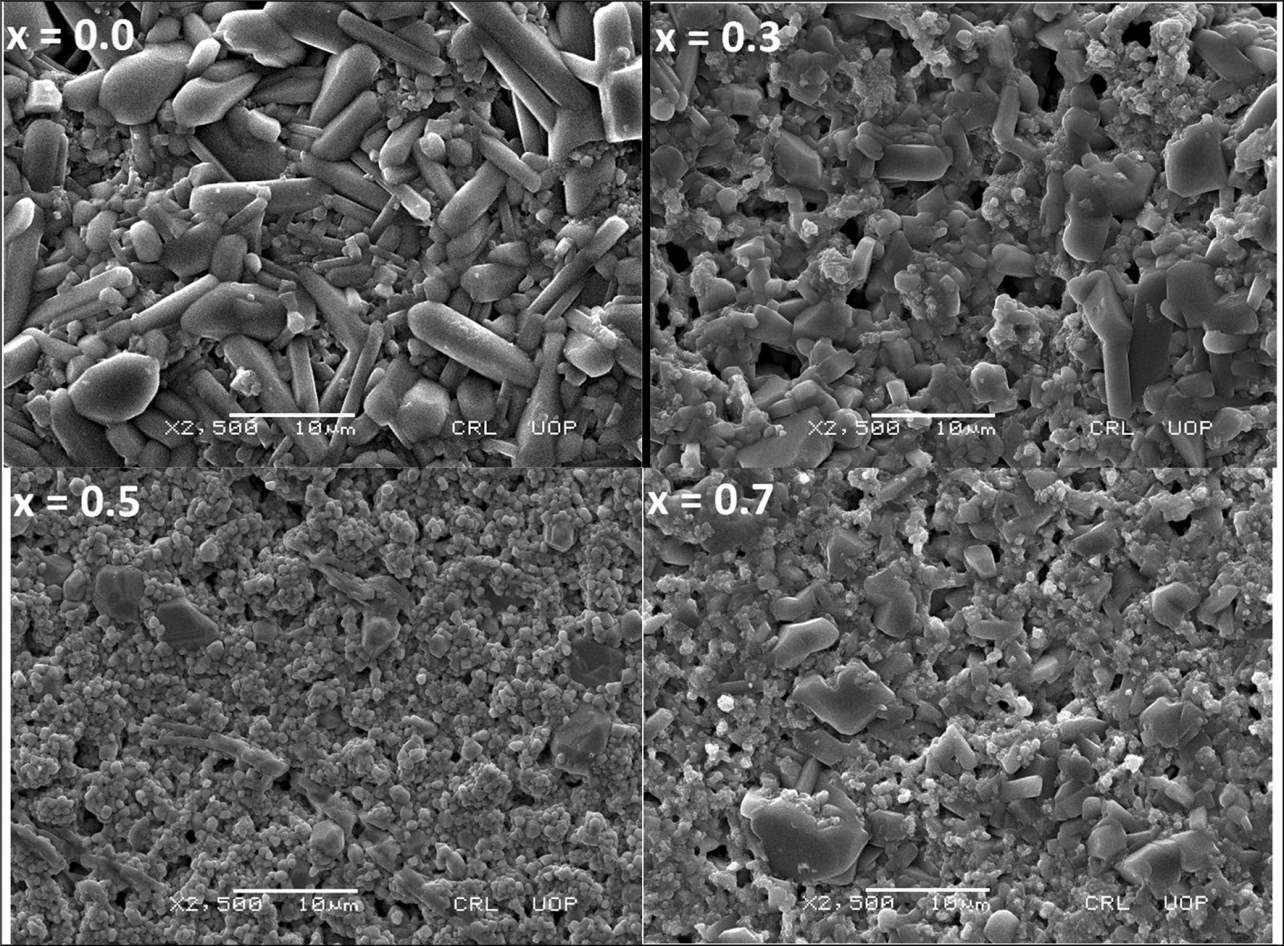
SEM Micrograph of Ba(Ti_1−x_Sn_x_)_4_O_9_, 0 ≤ x ≤ 0.7 ceramics.

SEM images were used to calculate the average grain size and density of Ba(Ti_1−x_Sn_x_)_4_O_9_, 0 ≤ x ≤ 0.7 ceramics (as shown in Fig. 2). It is clear from the figure that two types of surface morphologies are present in all samples (i.e. rod-like and spherical-like particles). The relative densities are increased, as the Sn^4^^+^ doping content increased in the base sample. The existence of cavities in the denser pellets confirms the existence of porosity. Thus, an increase in the relative density and decrease in the porosity was observed as the Sn content increased^20^. The porosity of these samples was calculated using Eq. (2) shown in Table 2.

2 $$ {\text{Porosity}} = \left( {\frac{{\rho_{th} - \rho_{ex} }}{{\rho_{th} }}} \right) \times 100\% $$

where ρ_th_ = theoretical density and ρ_ex_ = experimental density (calculated using Archimedes’ principle). The observed average grain size varies from ~ 10.7 to ~ 2 µm as dopant content x varies from 0.0 to 0.7. At x = 0.5, the Sn^4^^+^ doped BaTi_4_O_9_ system has a smaller average grain size of 0.8 µm with a more uniform grain size distribution. Thus, high densification (~ 99.4%) and low porosity (~ 0.55%) were achieved with Sn^4+^ = 0.5 content which reduces the growth of grains.

**Table 2:** Physical properties of Ba(Ti_1−x_Sn_x_)_4_O_9_, 0 ≤ x ≤ 0.7 sintered ceramics.

Composition (x)	ρ_th_ (gm/cm^3^)	ρ_ex_ (gm/cm^3^)	ρ_re_ (%)	ϵ_r_	Porosity (%)	Average grain size (µm)
0.0	5.9	4.5	76.3	30.08	23.72	10.7
0.3	4.953	4.48	90.5	37.57	9.55	6.45
0.5	5.611	5.58	99.4	57.29	0.55	2.54
0.7	5.347	5.25	98.2	46.63	1.81	5.56

### Optical properties

**Figure 3:**
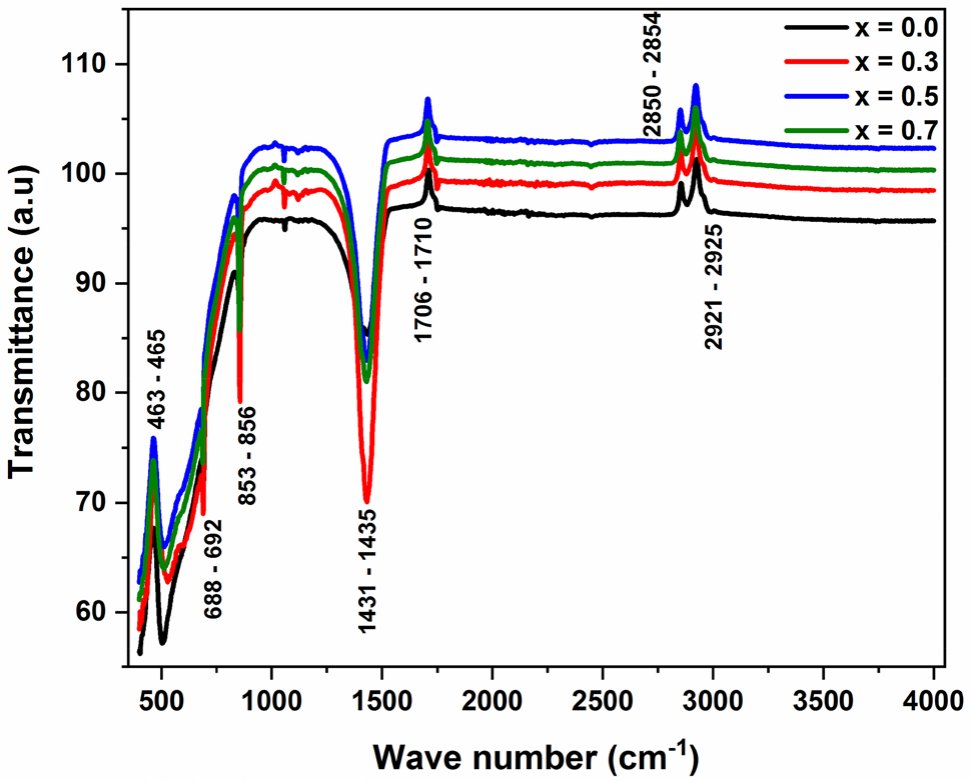
FTIR spectra of Ba(Ti_1−x_Sn_x_)_4_O_9_, 0 ≤ x ≤ 0.7 ceramics.

Figure 3 represents the FTIR spectra of Ba(Ti_1−x_Sn_x_)_4_O_9_, 0 ≤ x ≤ 0.7 ceramics at room temperature. The peaks that appear near ~ 2852, ~ 2922, and ~ 1433 cm^−1^ represents the symmetric, asymmetric, and bending vibrations of the H-C-H group, respectively^21^. Peaks appearing at ~ 854 cm^−1^ show the vibrational mode which relates to the stretching mode of the O-Ti–O system in the ceramic samples that confirmed the presence of BaTi_4_O_9_ structures^22^. The absorption mode appearing at ~ 690 cm^−1^ represents the Ti–O stretching mode of the octahedral group in complex perovskite structure^23,24^, as this mode appears only in BaTi_4_O_9_ ceramics. Furthermore, the structure of the BaTi_4_O_9_ ceramic sample has been confirmed by the XRD result.

**Figure 4:**
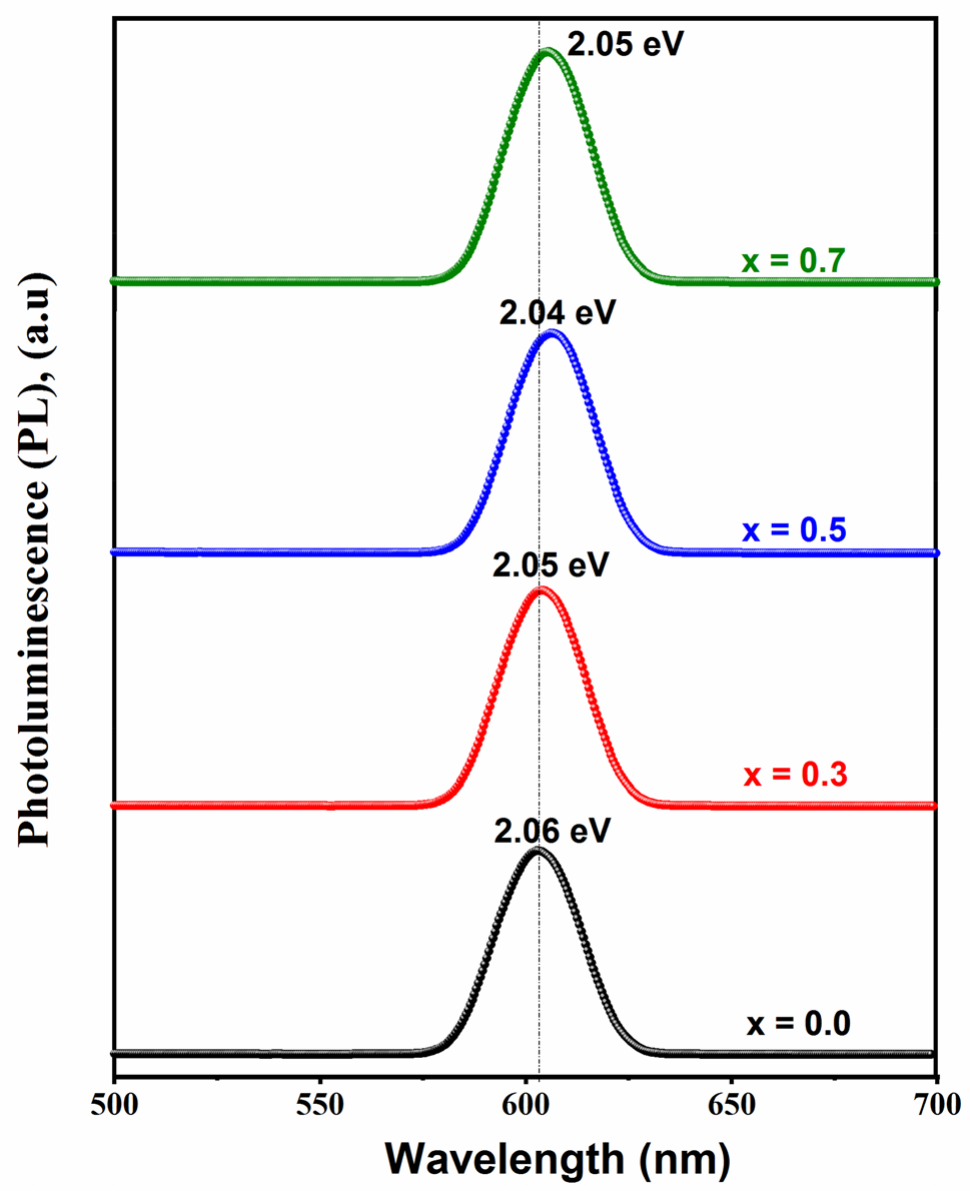
PL spectra of Ba(Ti_1−x_Sn_x_)_4_O_9_, 0 ≤ x ≤ 0.7 ceramics.

Photoluminescence spectroscopy of Ba(Ti_1−x_Sn_x_)_4_O_9_, 0 ≤ x ≤ 0.7 ceramics are shown in Fig. 4. The optical emission spectra are recognized as the recombination of electrons and holes in the state of transfer of carriers ions. By using Eq. (3), we have found the value of excitation energy of the samples.

3 $$  {\text{E}} = {\text{hc}}/\uplambda   $$

where E = optical excitation energy, h = Plank’s constant (~ 6.63 × 10^–34^ Js) c = velocity of light (3 × 10^8^ m/s) and λ is the emission wavelength. Emission at photoluminescence peak of the samples were recorded at ~ 603, 604, 606.5 and 605 nm with excitation energy ~ 2.06, 2.05, 2.04 and 2.05 eV for x = 0.0, 0.3, 0.5, and 0.7 content of Sn^4^^+^ dopant, respectively. Photoluminescence is a multi-photonic process that is an optical energy emission occurred in the optical range by many vibrational modes within the samples^25^. Within the energy band-gap, the photoluminescence process confirmed that due to localizing state the order/disorder structure may be affected directly. Hence, the structural order/disorder may be increased with increasing the energy band gap^26^. It was recorded that a broad-emission spectrum was located at ~ 604 nm and have an optical excitation energy (~ 2.06 eV) which was smaller than the energy band gap of extremely ordered BaTi_4_O_9_ ceramics located at ~ 558 nm (~ 2.23 eV) which may be due to the absence of oxygen vacancy^27^. In the photoluminescence spectrum, the red color may occur due to the oxygen vacancy.

### Microwave dielectric properties

**Figure 5:**
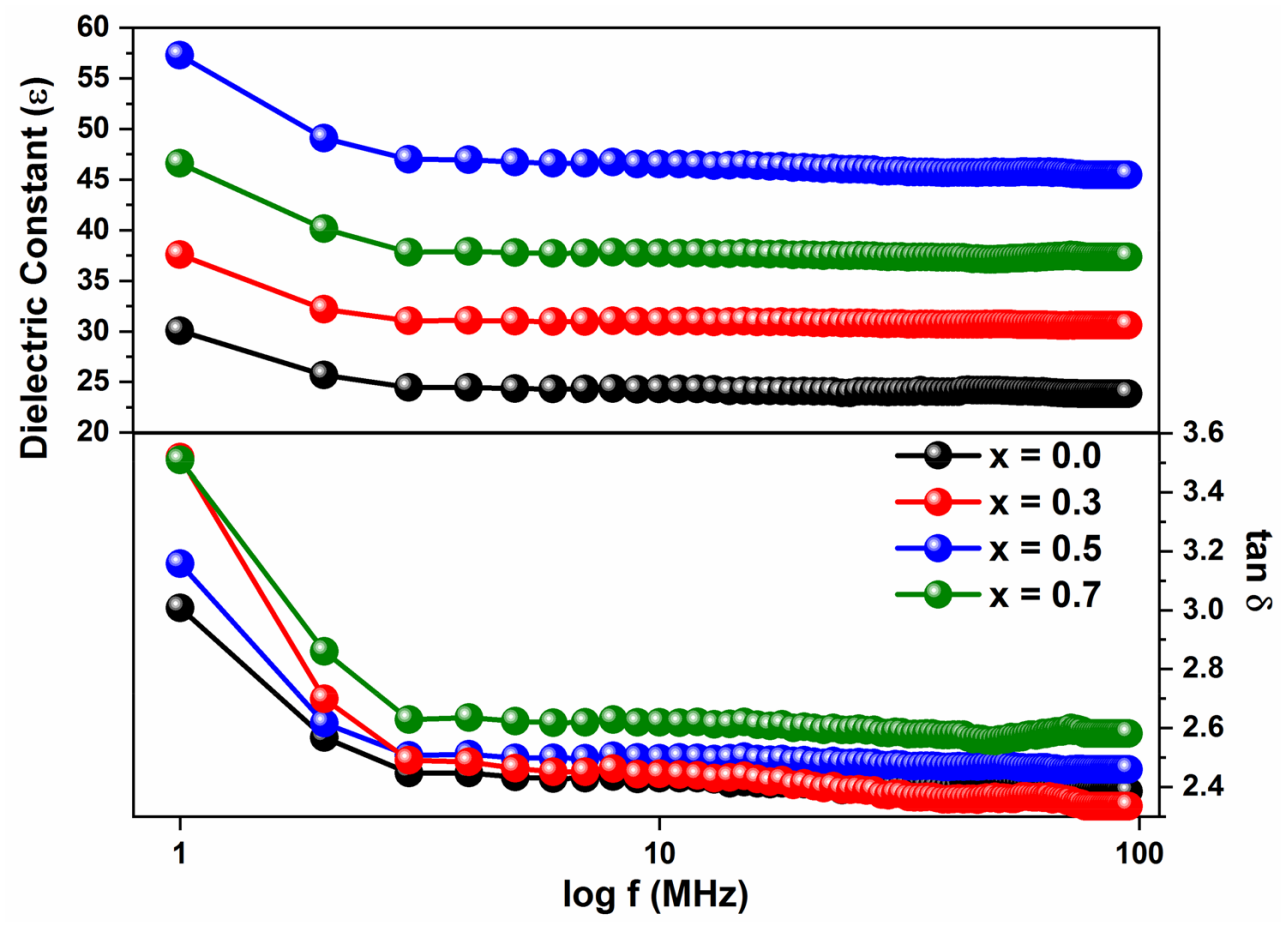
Frequency dependence of Dielectric constant (ε) and dielectric loss (tanδ) of Ba(Ti_1−x_ Sn_x_)_4_O_9_, 0 ≤ x ≤ 0.7 ceramics.

The variation of the relative permittivity (ε_r_) and tangent loss (tanδ) values of Ba(Ti_1−x_Sn_x_)_4_O_9_, 0 ≤ x ≤ 0.7 sintered ceramics versus frequency at room temperature is shown in Fig. 5. Due to increasing frequency, the values of ε_r_ and tanδ decrease exponentially in the samples. The high value of ε_r_ at resonant frequency (*f*_o_) can be described based on:

1. According to Maxwell–Wagner’s model, the dielectric materials are consist of fine conductive grains which are surrounded by grain boundaries. Large polarization is caused by the motion of charge carriers from grain to the grain surface.
2. The ionic polarization
3. The majority are due to crystal defects, vacancies, and grain defects etc^28,29^.

The increase in ε_r_ values with Sn^4+^ contents may be recognized by the substitution of a larger ionic radius of Sn^4+^ (~ 0.69 Å) cation for a smaller ionic radius of Ti^4+^ (~ 0.64 Å) cation^30^. To increasing the bond length of complex perovskite (i.e. AB_4_O_9_) the larger ionic radius cation may be substituted at B-site cation. The Sn^4+^ contents greatly affected the microwave dielectric properties due to high ionic polarization^31,32^. The maximum dielectric constant is obtained at x = 0.5 at maximum relative density (i.e. at low porosity). Because charge carriers need a medium to propagate and hence dielectric constant decreases with increasing material porosity^33,34^. Dielectric loss decreases with frequency due to the space charge polarization in all samples. At the lowest frequency, the maximum tangent loss occurs due to the presence of defects, impurities, and porosity in the ceramic samples^35^.

In general, the microwave dielectric properties of ceramics are dependent on intrinsic and extrinsic factors. The intrinsic properties are due to the interaction of materials phonons with the applied ac field. Thus the intrinsic properties also depend upon the crystal symmetry as observed in many single crystals^36^**.** The extrinsic properties are due to the imperfection in the crystal structure such as dopants or impurity atoms, grain boundaries, vacancies, order–disorder, secondary phases, etc^36–40^. Mostly, extrinsic factors are process-dependent and can be optimized. In this report, the sintering of these ceramic samples was done at a very high temperature (i.e. > 1300 °C for 4 h). The sintering at a high temperature may be causing the partial reduction of Ti^4+^ to Ti^3+^ ions. When Sn^4+^ is doped, it helps to maintain Ti^4+^ due to the following reaction:

$$ {\text{Sn}}^{{{4} + }} + {\text{ Ti}}^{{{3} + }} \rightleftharpoons {\text{Sn}}^{{{3} + }} + {\text{ Ti}}^{{{4} + }} $$

and, thus control the reduction of Ti^4+^ to Ti^3+^ ions.

Therefore, at high-temperature sintering, a sintered layer acts as a shield that prevents the transport of oxygen to the core. Due to the lack of oxygen at the core, oxygen vacancies or titanium interstitials are produced. The presence of vacancies in the lattice is responsible for damping of phonon modes and hence maybe leads to enhancement of dielectric properties and Q-factors^36,41,42^.

**Figure 6:**
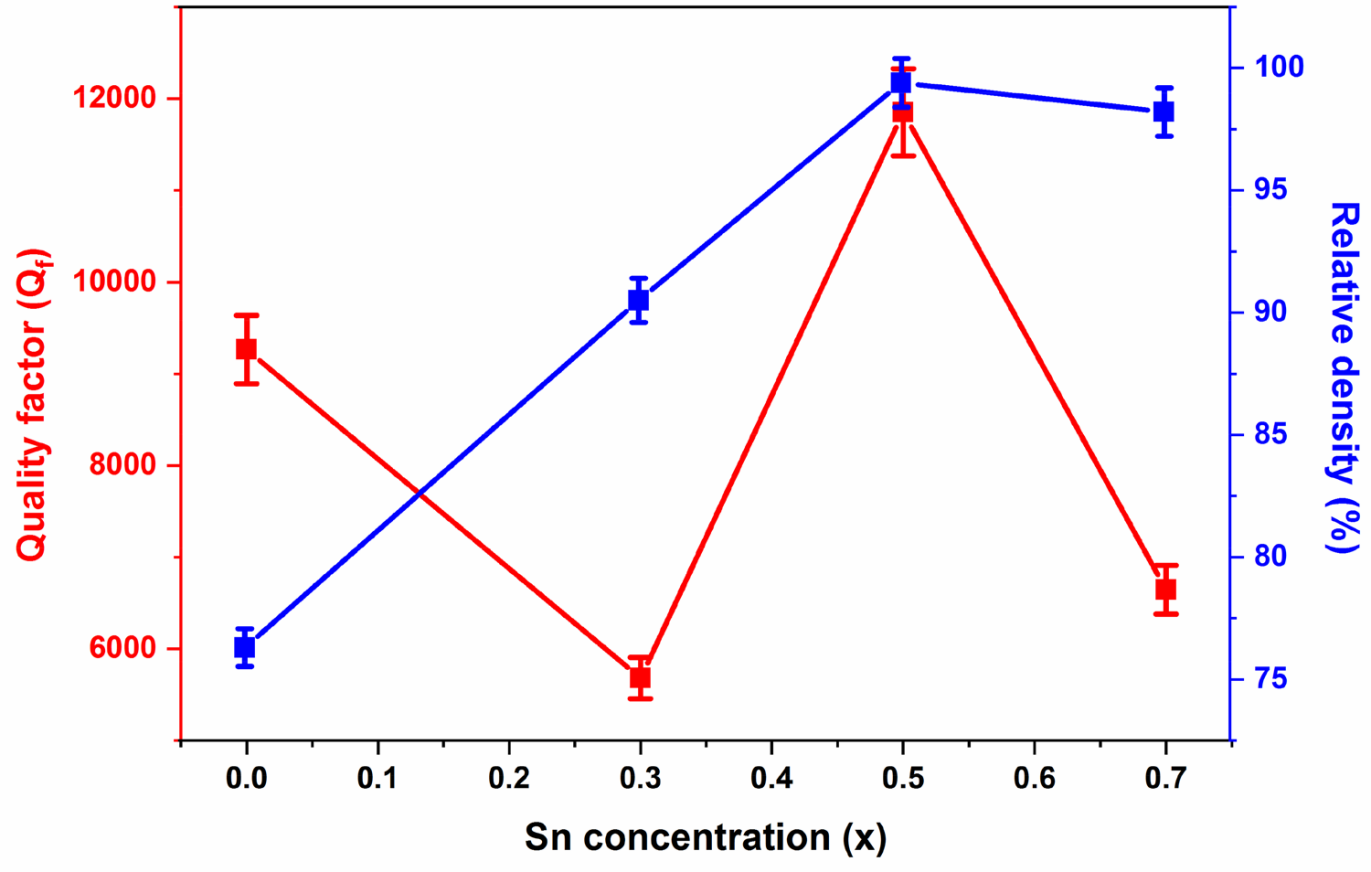
Variation of relative density and Quality factor (Q_f_) versus Sn^4^^+^ contents of Ba(Ti_1−x_Sn_x_)_4_O_9_, 0 ≤ x ≤ 0.7 ceramics.

The variation of quality factor (Q_*f*_) and relative density (%) of Ba(Ti_1−x_Sn_x_)_4_O_9_ sintered ceramics as a function of composition (x) is shown in Fig. 6. Initially, the quality factor decreases from 9264.49 to 5681.16 with increasing Sn^4+^ content (from 0 to 0.3). The observed decrease in the value of Q_*f*_ may be accepted due to the substitution of larger Sn^4+^ cation ion on the B-site cation, contributing to harmonic vibrational modes^43–45^ and another reason may be the phase transition. Additionally, an increase in Sn^4+^ content leads to a high Q_*f*_ value, which may occur due to: (1) The phonon modes of B-site harmonic, and (2) Relative density of the ceramic samples.

**Figure 7:**
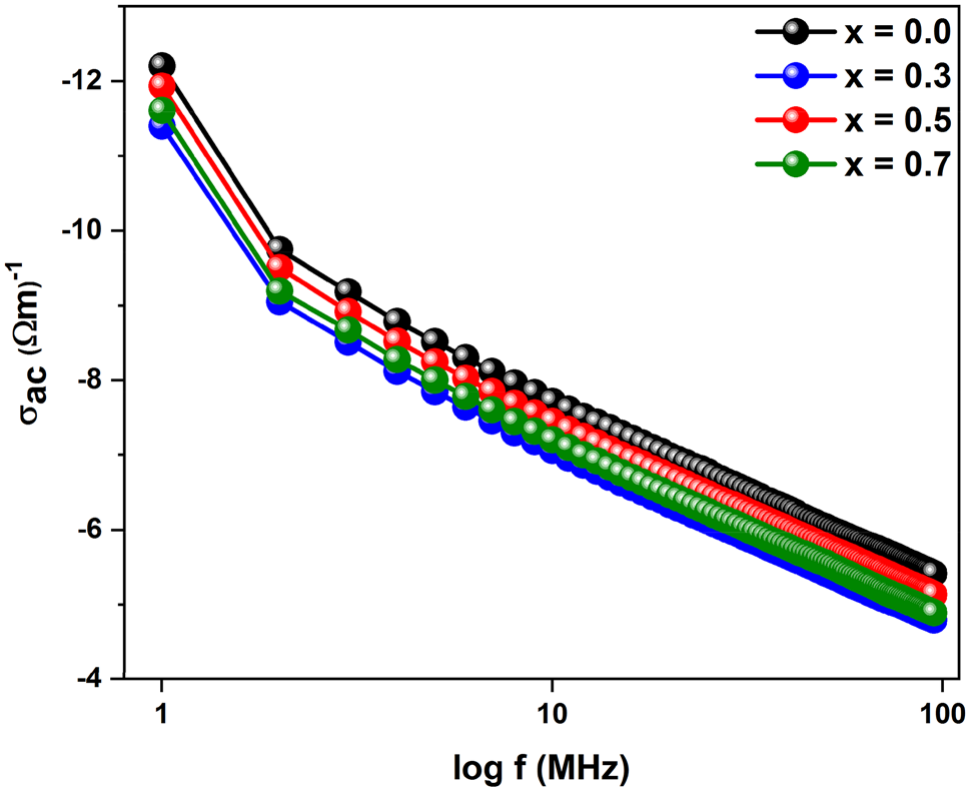
Variation of ac conductivity as a function of the frequency of Ba(Ti_1−x_Sn_x_)_4_O_9_, 0 ≤ x ≤ 0.7 ceramics.

The variation of ac conductivity of Ba(Ti_1−x_Sn_x_)_4_O_9_, 0 ≤ x ≤ 0.7 sintered ceramics versus frequency is shown in Fig. 7. It is clear from the graph that ac conductivity depends upon the frequency and does not show any significant variation at the lowest frequency. The maximum value of ac conductivity at a lower frequency may be due to the rising state of localization in the hopping process. By the application of electric field, the hopping frequency of the charge carrier increased which result in the highest value of ac conductivity towards the high-frequency region^46^.

## Conclusion

Solid solutions of Ba(Ti_1−x_Sn_x_)_4_O_9_, (0 ≤ x ≤ 0.7) ceramics were fabricated through a mixed-oxide route. The average crystallite size of these ceramic samples lies in the range of 30–90 nm with phase change from orthorhombic (space group = Pnmm) to Tetragonal (P4*mm*) structure. Sintered ceramics attained 99.5% of the theoretical density at content (x = 0.5) and fine grain growth with uniformity was achieved. Photoluminescence confirmed that the present state of localization within the band-gap may affect the structural order/disorder. The dielectric properties of sintered ceramic samples showed ε_r_ = 57.29, and high Q_f_ = 11,852. The increase in ac conductivity is due to the hopping mechanism. Based on the above-obtained results, these ceramic materials can be used for filter applications.

## Data Availability

The data of this study are available from the corresponding author upon reasonable request.
